# Q-Ball of Inferior Fronto-Occipital Fasciculus and Beyond

**DOI:** 10.1371/journal.pone.0100274

**Published:** 2014-06-19

**Authors:** Eduardo Caverzasi, Nico Papinutto, Bagrat Amirbekian, Mitchel S. Berger, Roland G. Henry

**Affiliations:** 1 Department of Neurology, University of California San Francisco, San Francisco, California, United States of America; 2 Joint Graduate Group in Bioengineering, University of California San Francisco and University of California Berkeley, San Francisco/Berkeley, California, United States of America; 3 Department of Neurological Surgery, University of California San Francisco, San Francisco, California, United States of America; Beijing Normal University, China

## Abstract

The inferior fronto-occipital fasciculus (IFOF) is historically described as the longest associative bundle in the human brain and it connects various parts of the occipital cortex, temporo-basal area and the superior parietal lobule to the frontal lobe through the external/extreme capsule complex. The exact functional role and the detailed anatomical definition of the IFOF are still under debate within the scientific community. In this study we present a fiber tracking dissection of the right and left IFOF by using a q-ball residual-bootstrap reconstruction of High-Angular Resolution Diffusion Imaging (HARDI) data sets in 20 healthy subjects. By defining a single seed region of interest on the coronal fractional anisotropy (FA) color map of each subject, we investigated all the pathways connecting the parietal, occipital and posterior temporal cortices to the frontal lobe through the external/extreme capsule. In line with recent post-mortem dissection studies we found more extended anterior-posterior association connections than the “classical” fronto-occipital representation of the IFOF. In particular the pathways we evidenced showed: a) diffuse projections in the frontal lobe, b) fronto-parietal lobes connections trough the external capsule in almost all the subjects and c) widespread connections in the posterior regions. Our study represents the first consistent in vivo demonstration across a large group of individuals of these novel anterior and posterior terminations of the IFOF detailed described only by post-mortem anatomical dissection. Furthermore our work establishes the feasibility of consistent in vivo mapping of this architecture with independent in vivo methodologies. In conclusion q-ball tractography dissection supports a more complex definition of IFOF, which includes several subcomponents likely underlying specific function.

## Introduction

Human cognition is supported by large-scale cortical and subcortical networks subserved by bundles of axons, connecting cortical and subcortical neurons. In the literature, white matter fibers are usually classified as commissural (between hemispheres), projection (between higher and lower brain and spinal cord centers) or association fibers (between cortical regions within the same hemisphere). Particularly the latter are subdivided into short and long association fibers.

The inferior fronto-occipital fasciculus (IFOF) is the longest associative bundle and it connects various parts of the occipital cortex, temporo-basal area and the superior parietal lobule to the frontal lobe [1]. The exact functional role of this bundle is controversial, however a possible role in language networks of semantic processing has been proposed [2]–[7]. After more than a century from its first descriptions (by Dejerine in 1895 and Curran in 1909) [8], the detailed anatomical definition of the IFOF is still under discussion within the scientific community; is it a distinct pathway or is it just a portion of a bigger bundle that includes the inferior longitudinal fasciculus (ILF) as well?

Post mortem anatomical dissection and in vivo diffusion MRI fiber tractography are current techniques that have enabled the segmentation of specific white matter fiber bundles and have been used to study the main course of the IFOF at the level of the insula and the temporal lobe [9]–[13]. Only recently, anatomical dissection studies have speculated about the anterior (frontal) [14]–[16] and posterior (occipital, parietal) [16], [17] cortical terminations of this fascicle; these studies seem to finally support the hypothesis of the IFOF being an independent bundle. However, these results have not been completely replicated by fiber tracking studies in part due to limitations in methods used so far.

Even though fiber tractography with the Diffusion Tensor Imaging (DTI) model has strongly contributed to our knowledge about fiber pathways, it suffers from several serious shortcomings in resolving extent, origin and cortical termination [18]. These shortcomings arise mainly because of partial volume effects [19] in voxels prone to tissues boundaries and regions where there are crossing or kissing fibers [20], [21]. Recently, tractography algorithms based on diffusion models that use High-Angular Resolution Diffusion Imaging (HARDI) have been developed. In particular constrained spherical deconvolution (CSD) [22], [23] and q-ball [18], [24] have shown to improve the ability to resolve white matter fiber tractography in crossing regions. By applying in vivo diffusion techniques, only two papers [15], [25] provide some description of the IFOF beyond its classical representation (a ventral associative pathway connecting the ventral occipital lobe and the orbitofrontal cortex) [13], [26]. The paper by Sarubbo et al. is mainly focused on post-mortem dissection of the IFOF in five subjects. Also included in this paper are results on a single case using DTI fiber tracking in vivo confirming novel frontal terminations, however no demonstration of the novel parietal terminations was provided [27]. A second recent work, using spherical deconvolution, investigated the IFOF based on the traditional understanding of the pathways [25]. Although they partially reported widespread anterior terminations, these are not described in details and no detection of the posterior terminations was reported.

The main goal of the present work was the fiber tracking dissection of the anatomy of the IFOF by using a q-ball residual-bootstrap reconstruction of a HARDI data-set in 20 healthy subjects, in order to 1) extend the in vivo diffusion MRI studies with a model suitable of overcoming the well-known DTI limitations, 2) evaluate a new tractography approach for the definition of the IFOF based on a simple single-subject region of interest (ROI) drawing.

## Materials and Methods

### MRI Data Acquisition

Research was performed in compliance to the Code of Ethics of the World Medical Association (Declaration of Helsinki) and the standards established by our Institution. The Committee on Human Research at the University of California, San Francisco (UCSF) approved the study protocol. Written informed consent was obtained from all participants.

The study cohort consisted of twenty subjects (11 male and 9 female; age 42±12). Handedness was confirmed by the Edinburgh Handedness Inventory scale. Fifteen patients out of twenty resulted right-handed, two left-handed and three with no dominance. All subjects had no history of psychiatric, neurological or cognitive impairment and provided written informed consent to participate in this study.

Subjects were scanned with a Signa 3T General Electric Medical Systems scanner. 3D high-resolution T1-weighted IRSPGR (TR/TE/TI  = 7/2/400 ms, 180 axial slices 1 mm thickness, 0.94×0.94 mm^2^ in plane resolution) and HARDI (TR/TE  = 6425/80 ms, 50 axial slices, 2.2 mm^3^ isotropic voxel, b-value = 2000 s/mm^2^, 55 diffusion gradients, 1 b0) datasets were acquired.

### MRI Data Analysis

HARDI datasets were corrected for movement and eddy-current distortions using FMRIB Software Library (FSL). The original gradient table was consequently rotated [28]. Diffusion Imaging in Python (Dipy) was used to estimate fractional anisotropy (FA) and for q-ball residual-bootstrap fiber tracking [24]. For each subject, the FA maps were co-registered to the anatomical images and to the FSL 2×2×2 mm^3^ resolution Montreal Neurological Institute (MNI) atlas using FSL linear and non-linear transformations (FMRIB’s FLIRT and FNIRT registration tools).

### Tracking Methods

A single plane ROI was defined on the coronal FA color map of each subject. The selected coronal plane was the one passing through the anterior commissure, on which it is easy to select the anterior-posterior (green coded) tracks of the left external/extreme capsule. At this level the coherence and anisotropy of the expected bundles is very high and easily identified. In each subject the seed ROI was defined in order to fully cover this region and a couple of voxels at the margin. The average dimension of the seed ROIs was around 15×15 mm^2^. The signal was fit to spherical harmonics of even orders up to 4 and orientation distribution functions (ODFs) with constant solid angle factor were estimated from the data using the methods of Tristan-Vega et al. [29], [30]. Residual-bootstrap tractography [24] was applied by computing bootstrap ODFs at each voxel and extracting peak detection from these ODFs. In order to identify the primary fiber orientation, ODF peaks were further refined by excluding peaks that were less than 45° from a larger peak and small peaks with values less than. 25 times the maximum of the ODF. The principle fiber orientations from the bootstrap ODFs provided the distribution of fiber tracking directions. Residual-boostrap fiber tracking was initiated at the external capsule ROI with 11^3^ seed points per voxel uniformly distributed across the three dimensions.

Tracking was regulated using an FA threshold = 0.15 and max angle = 60° as stopping parameters in the algorithm [31]. The results were visualized with Trackvis (http://trackvis.org). In order to select the posterior termination of the IFOF, a coronal plane perpendicular to the ac-pc line was applied at the level where the parieto-occipital and calcarine sulci meet. A careful inspection of results was performed by a neuroradiologist with 8 years experience (EC): firstly we excluded the presence of tracking artifact such as streamlines creating loops or passing through several GM regions; secondly we excluded that susceptibility distortions may have affected the outcome of the tracking [32]. In all subjects, streamlines were far from regions in general interested by susceptibility distortions, such as the temporal lobes and other regions near the paranasal sinuses.

Cortical gray matter segmentation and parcellation was performed with Freesurfer (Desikan-Kyliany Atlas) in order to obtain volumes of interest (VOIs) in the T1-weighted anatomical scans native-space [33]–[35]. Segmentation results were visually assessed for accuracy by a trained operator (EC). For each subject, frontal, occipital and parietal VOIs were transformed to the native diffusion space using the inverse of the computed transformations and growth to include 2 mm of underlying white matter. These VOIs were used to classify independently the different terminations (cortical endpoints) both anteriorly in the frontal and posteriorly in the parietal, occipital lobes and more caudal portion of the temporal lobes. The results are summarized in Table 1 and Table 2, where we report the percentage of subjects in which the IFOFq appears to reach each VOI. In a further step we also combined the middle frontal (rostral and caudal portions), inferior frontal (pars opercularis, triangularis, orbitalis) and orbitofrontal (medial and lateral) VOIs in order to compare our results to the ones reported by Sarubbo et al. [15]. Besides an exhaustive post mortem dissection of the IFOF, their paper furnished for the first time a detailed description of IFOF frontal terminations by using a diffusion model (DTI) on a single subject. Our purpose was to extend these results on a group of 20 subjects by using q-ball, in order to get more reliable results.

**Table 1:** IFOFq anterior segmentation results.

(A) Lefthemisphere			Frontal lobe										
			Caudalmiddle	lateralorbito	medialorbito	paracentral	Pars opercularis	Parsorbitalis	Parstriangularis	precentral	rostralmiddle	superior	Frontalpole
1	**Y**		0	0	0	0	**1**	**1**	**1**	0	**1**	**1**	**1**
2	**Y**		0	**1**	**1**	0	**1**	**1**	**1**	0	**1**	**1**	**1**
3	**Y**		**1**	0	0	0	**1**	**1**	**1**	0	**1**	**1**	**1**
4	**Y**		0	**1**	**1**	0	0	**1**	**1**	0	**1**	**1**	**1**
5	**Y**		0	**1**	**1** *	0	**1**	**1**	**1**	0	**1**	0	0
6	**Y**		0	**1**	**1**	0	**1** *	**1**	**1**	0	**1**	**1**	**1**
7	**Y**		0	**1**	**1** *	0	**1**	**1**	**1**	0	**1**	**1**	**1** *
8	**Y**		0	**1**	**1** *	0	**1**	**1**	**1**	0	**1**	**1**	**1**
9	**Y**		0	0	0	0	**1**	**1**	**1**	0	**1**	**1**	**1** *
10	**Y**		**1** *	**1**	**1**	0	**1** *	**1**	**1**	0	**1**	**1**	**1**
11	**Y**		0	**1**	**1** *	0	**1**	**1**	**1**	0	**1**	**1**	**1**
12	**Y**		0	**1**	**1**	0	0	**1**	**1**	0	**1**	**1**	**1**
13	**Y**		0	**1**	0	0	**1**	**1**	**1**	0	**1**	**1**	**1** *
14	**Y**		0	**1**	**1**	0	0	**1**	**1**	0	**1**	**1**	**1**
15	**Y**		0	**1**	**1**	0	**1** *	**1**	**1**	0	**1**	**1**	**1** *
16	**Y**		0	**1**	0	0	**1** *	**1**	**1**	0	**1**	**1**	**1**
17	**Y**		0	**1**	**1**	0	0	**1**	**1**	0	**1**	**1**	**1**
18	**Y**		0	**1**	**1**	0	0	**1**	**1**	0	**1**	**1**	**1**
19	**Y**		0	**1**	**1**	0	**1**	**1**	**1**	0	**1**	**1**	**1**
20	**Y**		0	**1**	**1**	0	**1**	**1**	**1**	0	**1**	**1**	**1** *
**% of 1**	**100.00%**		**10.00%**	**85.00%**	**75.00%**	**0.00%**	**75.00%**	**100.00%**	**100.00%**	**0.00%**	**100.00%**	**95.00%**	**95.00%**
**(B) Right hemisphere**			**Frontal lobe**										
			**Caudal** **middle**	**lateral orbito**	**medial orbito**	**paracentral**	**Pars opercularis**	**Pars orbitalis**	**Pars triangularis**	**precentral**	**rostral middle**	**superior**	**Frontal pole**
1		**Y**	0	**1**	**1**	0	**1**	**1**	**1**	0	**1**	**1**	0
2		**Y**	0	**1**	**1**	0	**1**	**1**	**1**	0	**1**	**1**	**1**
3		**Y**	0	**1**	**1**	0	**1** *	**1**	**1**	0	**1**	**1**	**1**
4		**Y**	0	**1**	**1**	0	**1**	**1**	**1**	0	**1**	**1**	**1**
5		**Y**	0	**1**	0	0	0	**1**	**1**	0	**1**	**1** *	0
6		**Y**	0	**1**	0	0	0	**1**	**1**	0	**1**	**1**	**1**
7		**Y**	0	**1**	**1** *	0	**1**	**1**	**1**	0	**1**	**1** *	**1** *
8		**Y**	0	**1**	**1** *	0	**1**	**1**	**1**	0	**1**	**1**	**1**
9		**Y**	0	**1**	0	0	**1** *	**1**	**1**	0	**1**	**1**	**1**
10		**Y**	0	**1**	**1**	0	**1**	**1**	**1**	0	**1**	**1**	0
11		**Y**	0	**1**	0	0	0	**1**	**1**	0	**1**	**1**	**1**
12		**Y**	0	**1**	**1**	0	0	**1**	**1**	0	**1**	**1**	**1**
13		**Y**	**1** *	**1**	0	0	**1**	**1**	**1**	0	**1**	**1**	**1**
14		**Y**	**1** *	**1**	0	0	**1** *	**1**	**1**	0	**1**	**1** *	**1** *
15		**Y**	0	**1**	**1** *	0	0	**1**	**1**	0	**1**	**1**	**1** *
16		**Y**	0	**1**	0	0	**1**	**1**	**1**	0	**1**	**1** *	**1**
17		**Y**	0	**1**	**1**	0	0	**1**	**1**	0	**1**	**1** *	**1**
18		**Y**	0	**1**	0	0	**1** *	**1**	**1**	0	**1**	**1**	**1**
19		**Y**	0	**1**	**1**	0	0	**1**	**1**	0	**1**	**1**	**1**
20		**Y**	0	**1** *	0	0	**1** *	**1**	**1**	0	**1**	**1**	**1**
**% of 1**		**100.00%**	**10.00%**	**100.00%**	**55.00%**	**0.00%**	**65.00%**	**100.00%**	**100.00%**	**0.00%**	**100.00%**	**100.00%**	**85.00%**

Single subject exploration of the left (A) and right hemispheres (B) IFOFq in 20 subjects based on the frontal lobe terminations. Freesurfer was used to parcellate frontal cortical gray matter into volumes of interest (11 VOIs). These VOIs were used to characterize the frontal termination of the IFOFq. For each hemisphere, the percentage of subjects in which each single VOI is interested by the IFOFq is reported on the bottom of the table.

*cases in which very few streamlines reached the VOI.

**Table 2:** IFOFq posterior segmentation results.

(A) Left hemisphere		Temporal lobe	Parietal lobe		Occipital lobe			
		Fusiform (caudal portion)	Angular gyrus	Superior parietal	Lingual	Pericalcarine	Lateral occipital	Cuneus
1	**Y**	**1**	0	**1**	**1**	**1**	**1**	0
2	**Y**	**1**	**1**	**1**	**1**	**1**	**1**	**1**
3	**Y**	0	**1**	**1**	**1**	**1**	**1**	0
4	**Y**	0	**1**	**1**	**1** *	**1**	**1**	0
5	**Y**	0	0	**1**	**1**	**1**	**1**	0
6	**Y**	**1**	**1**	**1**	**1**	**1**	**1**	0
7	**Y**	0	**1** *	**1**	**1**	**1**	**1**	0
8	**Y**	**1**	**1**	**1**	**1**	**1**	**1**	0
9	**Y**	**1**	**1**	**1**	**1**	**1**	**1**	0
10	**Y**	0	**1**	**1**	**1**	**1**	**1**	**1** *
11	**Y**	0	**1**	**1**	**1**	**1**	**1**	**1** *
12	**Y**	0	**1**	**1**	**1**	**1**	**1**	0
13	**Y**	**1**	**1**	**1**	**1**	**1**	**1**	**1**
14	**Y**	**1**	**1**	**1**	**1**	**1**	**1**	0
15	**Y**	**1**	**1**	**1**	**1** *	**1**	**1**	0
16	**Y**	**1**	**1**	**1**	**1**	**1**	**1**	**1**
17	**Y**	**1**	**1**	**1**	**1**	**1**	**1**	0
18	**Y**	0	**1**	**1**	**1**	**1**	**1**	**1** *
19	**Y**	**1** *	**1**	**1**	**1**	**1**	**1**	0
20	**Y**	0	**1**	**1**	**1**	**1**	**1**	**1**
**% of 1**	**100.00%**	**55.00%**	**90.00%**	**100.00%**	**100.00%**	**100.00%**	**100.00%**	**35.00%**
**(B) Right hemisphere**		**Temporal lobe**	**Parietal lobe**		**Occipital lobe**			
		**Fusiform (caudal portion)**	**Angular gyrus**	**Superior parietal**	**Lingual**	**Pericalcarine**	**Lateral occipital**	**Cuneus**
1	**Y**	0	**1**	**1**	**1**	**1**	**1**	**1**
2	**Y**	**1**	**1**	**1**	**1**	**1**	**1**	**1**
3	**Y**	0	**1**	**1**	**1**	**1**	**1**	**1**
4	**Y**	**1** *	**1**	**1**	**1**	**1**	**1**	**1** *
5	**Y**	**1**	0	**1**	**1**	**1**	**1**	0
6	**Y**	**1**	**1**	**1**	**1**	**1**	**1**	**1**
7	**Y**	**1** *	**1**	**1**	**1**	**1**	**1**	**1**
8	**Y**	0	**1**	**1**	**1**	**1**	**1**	**1**
9	**Y**	**1**	**1**	**1**	**1**	**1**	**1**	**1**
10	**Y**	0	**1**	**1**	**1**	**1**	**1**	0
11	**Y**	0	**1**	**1**	**1**	**1**	**1**	**1**
12	**Y**	0	**1**	**1**	**1**	**1**	**1**	**1**
13	**Y**	**1**	**1**	**1**	**1**	**1**	**1**	0
14	**Y**	**1**	**1**	**1**	**1**	**1**	**1**	0
15	**Y**	0	**1**	**1**	**1**	**1**	**1**	0
16	**Y**	**1** *	**1**	**1**	**1**	**1**	**1**	**1**
17	**Y**	0	**1**	**1**	**1**	**1**	**1**	**1**
18	**Y**	**1** *	**1**	**1**	**1**	**1**	**1**	0
19	**Y**	**1**	**1**	**1**	**1**	**1**	**1**	0
20	**Y**	0	**1**	**1**	**1**	**1**	**1**	0
**% of 1**	**100.00%**	**55.00%**	**95.00%**	**100.00%**	**100.00%**	**100.00%**	**100.00%**	**60.00%**

Single subject exploration of the left (A) and right hemispheres (B) IFOFq in 20 subjects based on the posterior terminations in the parietal (P), occipital (O) and temporal (T) lobes. Freesurfer was used to parcellate P, O, T cortical gray matter into volumes of interests (VOIs). These VOIs were used to characterize the posterior terminations of the IFOFq. The table shows only the VOIs reached by the IFOFq. For each hemisphere, the percentage of subjects in which each single VOI is interested by the IFOFq is reported on the bottom of the table. All the occipital lobes VOIs showed (in a different degree) to be reached by posterior terminations of the IFOFq. In the temporal and parietal lobes only the caudal portion of the fusiform (T), the angular gyrus and the superior parietal gyrus (P) showed to be connected to the IFOFq.

*cases in which the particular VOIs were interested by very few streamlines.

### MNI Group Results in the MNI Space

Streamline density maps were computed in the subjects’ native space and thresholded to obtain binary masks containing all voxels that were visited by at least one streamline in the residual bootstrap tractography. These masks were spatially normalized to the MNI space using the linear and non-linear transformations computed for the co-registration of the FA maps to the FSL MNI atlas. The masks of the different subjects in the MNI space corresponding to the various tracks or sub-components were summed and normalized to obtain percentage probabilistic maps. A 3D rendering of the different tracks was obtained selecting an inferior threshold of 20% on the probabilistic maps using the FSL viewer. The 2×2×2 mm^3^ resolution MNI atlas was segmented and parcellated analogously to what was done for the T1-weighted images and used to classify the different subcomponents of the IFOF on a group basis.

## Results

**Figure 1:**
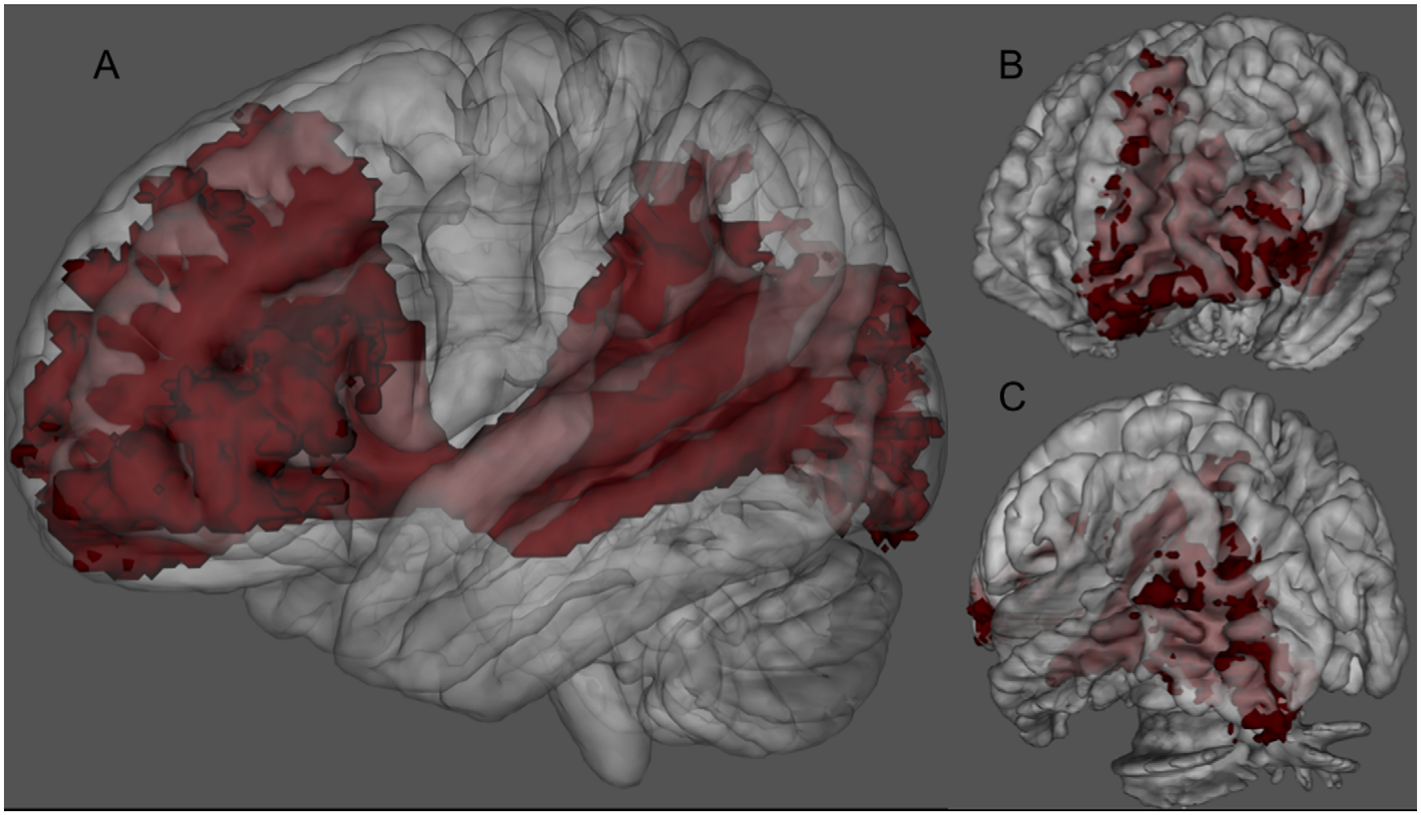
3D rendering of the left IFOFq. The left IFOFq, reconstructed on the 20 subjects and normalized to the MNI space, is visualized from a lateral (A), anterior (B) and posterior (C) point of view. In A the path of the whole bundle can be appreciated, whereas B and C show its group average cortical terminations. Voxels that were visited in the residual bootstrap tractography in at least 20% of subject are shown.

**Figure 2:**
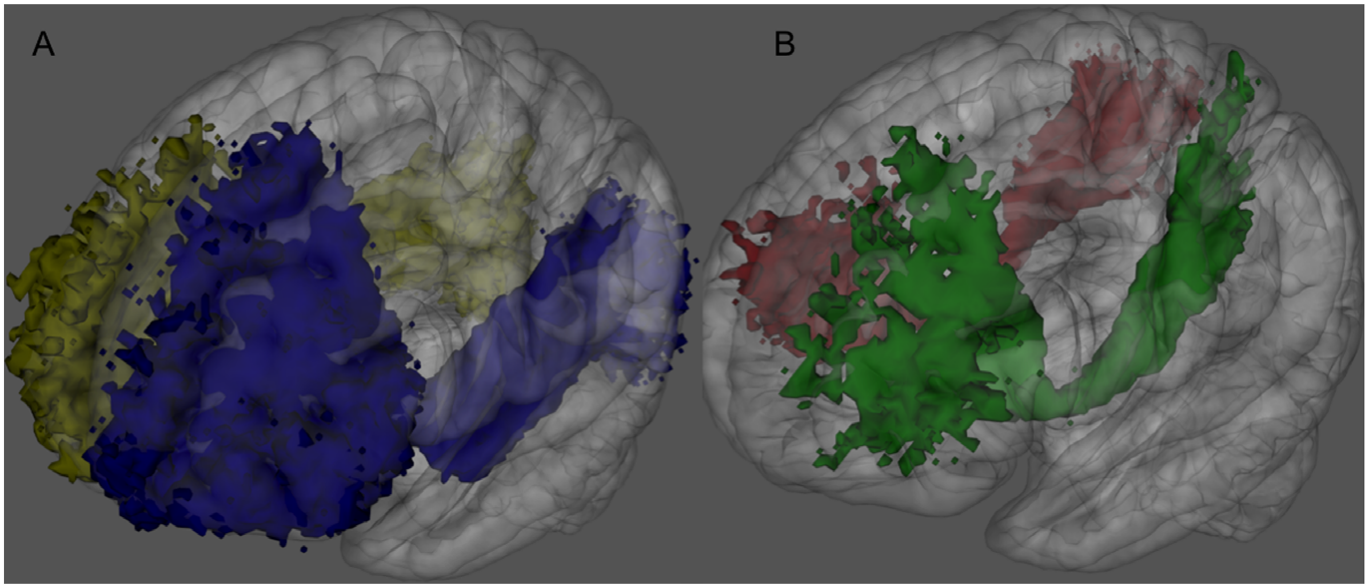
IFOFq subdivision into portions connecting frontal regions to either the occipital (A) or the parietal lobes (B). 3D rendering of the left (blue) and right (yellow) IFOFocc reconstructed on the 20 subjects normalized to the MNI space (A). 3D rendering of the left (green) and right (red) portions of the IFOFq connecting frontal and parietal lobes reconstructed on the 20 subjects normalized to the MNI space (B). Voxels that were visited in the residual bootstrap tractography in at least 20% of subject are shown.

**Figure 3:**
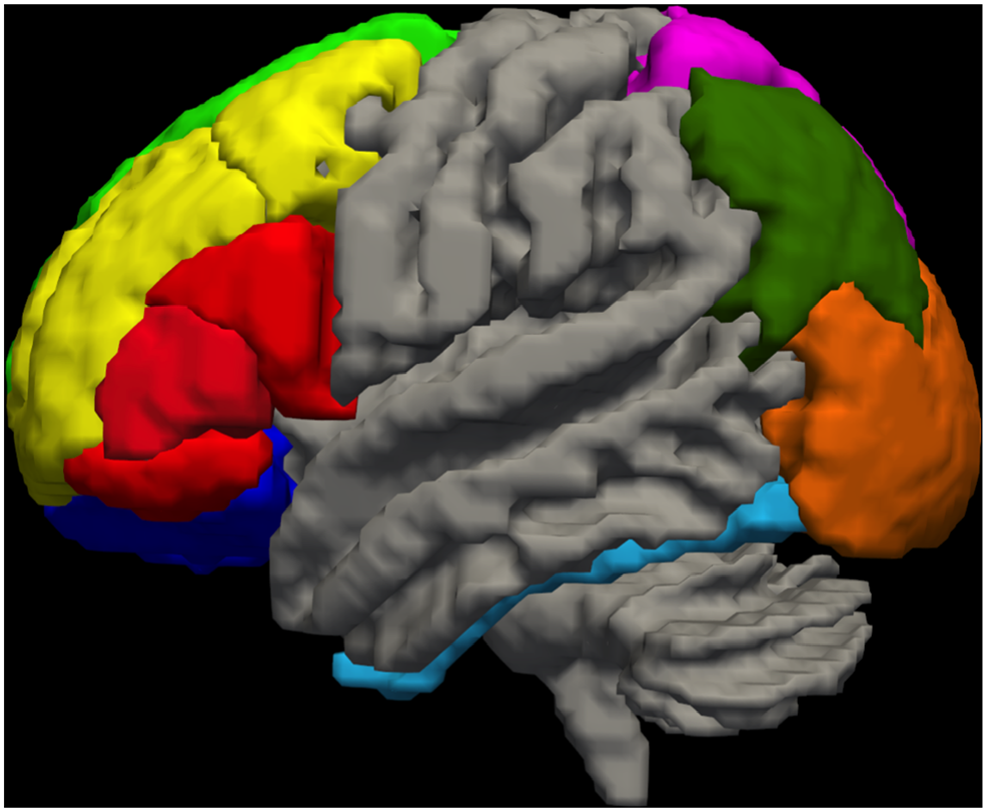
Schematic representation of the cortical regions connected by the IFOFq. 3D schematic representation in the MNI space of the Freesurfer-based regions, which showed connections through the IFOFq (left lateral view). Light green: superior frontal; yellow: caudal and rostral portions of middle frontal cortex; red: pars operularis, triangularis and orbitalis of the inferior frontal cortex; blue: lateral and medial portions of the orbitofrontal cortex; pink: superior parietal gyrus; green: angular gyrus; orange: occipital lobe; teal: fusiform gyrus. Fusiform gyrus was reached by IFOF termination only in its very caudal portion.

The IFOF was successfully identified using q-ball in all the subjects. The resulted tracks were more extended than the “classical” fronto-occipital representation of the IFOF (Fig. 1). For clarity we will refer to IFOFq when considering the full bundle we were able to track with the q-ball residual-bootstrap approach, whereas to IFOFocc for the “classical” IFOF (Fig. 2-A). The IFOFq showed a) diffuse projections in the frontal lobe, b) fronto-parietal lobes connections trough the external capsule in almost all the subjects (Fig. 2-B), and c) widespread connections in the posterior regions. Figure 3 shows a 3D schematic rendering in the MNI space of Freesurfer-based regions connected through the IFOFq.

### Frontal Termination of the IFOFq

**Figure 4:**
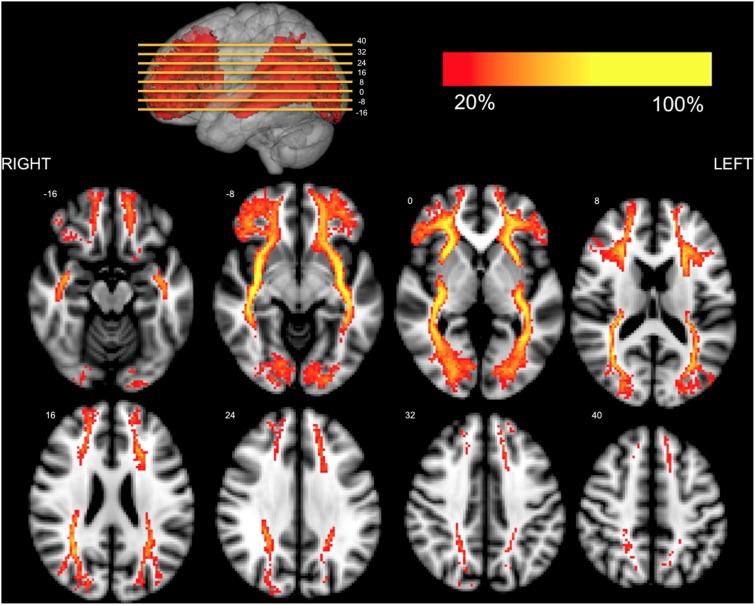
Axial representation of the IFOFq. The left and right IFOFq were reconstructed on 20 subjects and normalized to the MNI space (radiological orientation). Colors represent the percentage of subjects (from 20% to 100%) in which a voxel was visited in the residual bootstrap tractography. The MNI coordinates of the selected axial slices are reported in the 3D rendering on the top.

**Figure 5:**
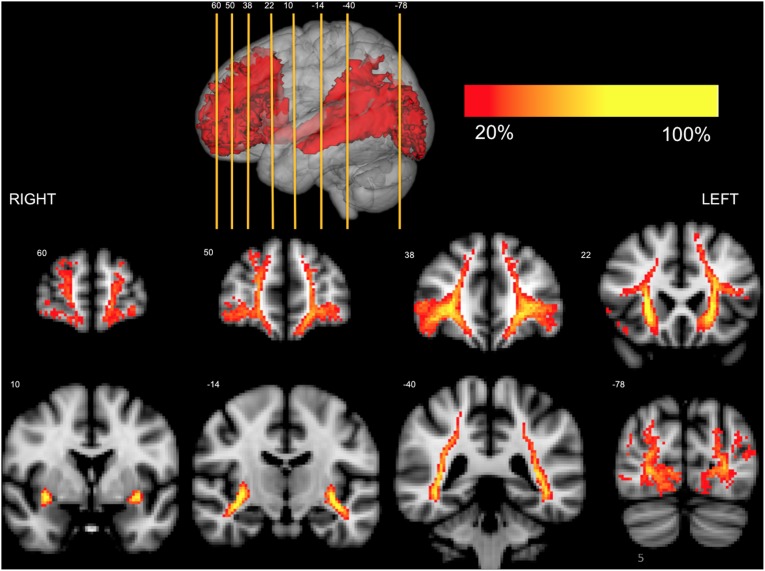
Coronal representation of the IFOFq. The left and right IFOFq were reconstructed on the 20 subjects and normalized to the MNI space (radiological orientation). Colors represent the percentage of subjects (from 20% to 100%) in which a voxel was visited in the residual bootstrap tractography. The MNI coordinates of the selected coronal slices are reported in the 3D rendering on the top.

**Figure 6:**
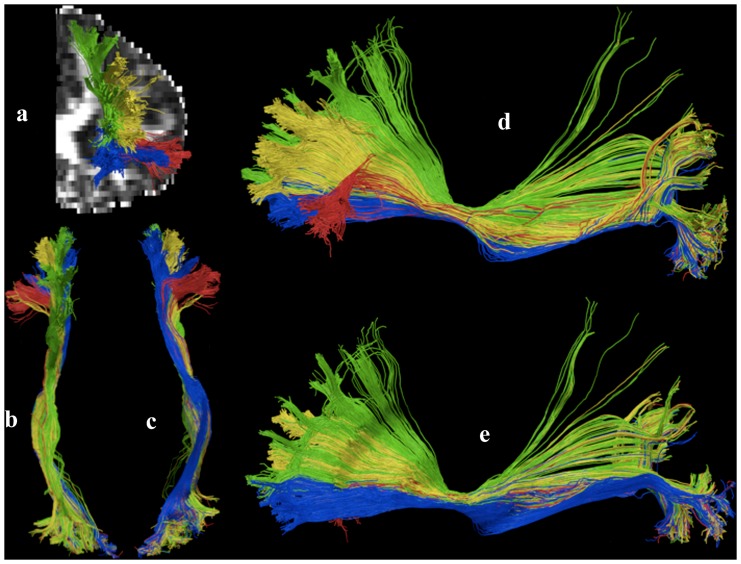
Anterior terminations of the IFOFq. Detailed description of the anterior terminations of the left IFOFq found in one illustrative subject. The colors of the subcomponent are consistent with the frontal VOIs color-code of Figure 3 and with Figure 13 by Sarubbo et al. Red: superficial layer; green: posterior terminations of the deep layer; yellow: middle terminations of the deep layer; blue: anterior terminations of the deep layer. a) anterior view; b) superior view; c) inferior view; d) lateral view; e) mesial view.

Fibers we identified spread towards different portion of the frontal region when entering the frontal lobe (Fig. 4–5). Based on the description by Sarubbo et al. we could organize these terminations in 4 main groups: 1) orbito-frontal region (lateral and medial orbitofrontal); 2) inferior frontal (pars orbitalis Broadman area (BA) 47 and triangularis BA 45 mainly); 3) rostral portion of the middle frontal (BA 10, BA46); 4) superior frontal gyrus (BA 8 BA 9) (Group results are summarized in Table 1; Fig. 6 shows a detailed example).

### Posterior Termination of the IFOFq

At the level of the external/extreme capsule IFOFq fibers were gathered together. As they entered the temporal stem they spread and project to lingual, pericalcarine and lateral occipital cortices (bilaterally true in all cases), cuneus (60% of subjects in the right hemisphere and 35% in the left hemisphere, see Table 2) and in the caudal portion of the fusiform gyrus (55% of subjects bilaterally). The anatomy of the IFOFocc projecting pathway was consistent across all subjects with greater than 75% overlap along its entire course. We also found parietal projections of the IFOFq mainly to the angular gyrus (90% of subjects for the left hemisphere and 95% for the right hemisphere) and superior parietal gyrus. The latter appeared to stand and run above the rest of the IFOFq, with the most frequent projection over the posterior portions of the superior frontal lobe (pre supplementary motor area). The parietal projecting pathway showed a greater inter-subject variability with a maximum of 66% overlap in its central part. Both classical IFOFocc and more parietal components of the IFOFq were in different extent contributing to its different frontal subcomponent (Fig. 4–5).

## Discussion

Q-ball residual-bootstrap reconstruction of the IFOF was successful in all subjects. Compared to the “classical” IFOF usually described in literature, we have found a more complex IFOF anatomy both rostrally in the frontal lobe as caudally (occipital, parietal and temporal lobes). IFOF function and anatomy are still matter of debate. Recent findings have raised questions about the detailed cortical connectivity of the IFOF. Direct cortical stimulation results in brain tumor patients suggested IFOF might contribute to language functions and might represent a crucial pathway of the ventral semantic stream [3], [36].

Since last century (Dejerine 1895) anatomists have formulated a classical definition for the IFOF as an association pathway between ventro-occipital and orbitofrontal regions. In vivo tractography (DTI) results reported in DTI atlases of human brain white matter pathways, have corroborated this interpretation [13]. However improvements in post mortem anatomical dissection techniques highlighted a more complex IFOF connectivity between premotor, prefrontal, occipito-parietal and basal temporal regions [15], [16]. These recent findings exposed the limitation of the DTI application in resolving high complexity cross-fiber areas and in showing the real cortical termination of white matter bundles. Diffusion models such as q-ball are ideal candidate for an extensive in vivo exploration and understanding of the IFOF anatomy.

For the first time we confirmed in vivo all the previously mentioned novel post-mortem results in a group of 20 healthy subjects with an up to date tractography algoritm such as q-ball. The anatomical dissection approach described by Martino et al. in 14 subjects was able to identify IFOF terminations both within and outside the occipital lobes. Our results are consistent with their anatomical description. In all our subjects there were occipital terminations in both the hemispheres especially towards pericalcarine and lateral occipital cortex. In contrast with Martino’s et al. manuscript there were also terminations towards the lingual gyrus, which they reported being reached by only optic radiation terminations. Due to their proximity and parallel course IFOF and optic radiation [37] are difficult to be resolved at the usual diffusion weighted imaging resolutions. Thus our results might reflect a failure in differentiating the two bundles. Surprisingly we found fewer terminations towards the cuneus (in 30% of patients in the left and 60% in the right hemisphere).

In line with Martino et al. findings there were posteriorly extra occipital terminations towards temporal and parietal lobes [16]. IFOFq showed temporal terminations, only in the posterior portion of the fusiform gyrus (bilaterally in 55% of subjects). This area is considered to be involved in verbal and facial recognition [38]. There were no connections to the inferior temporal gyrus. Two distinct regions of the parietal lobe were reached by the IFOFq: in all the subjects there were bilateral IFOFq terminations towards the superior parietal and angular giri. As the IFOFq is approaching the occipital lobe a portion of it directs laterally and superiorly towards the angular gyrus, the inferior portion of parietal lobe at the edge with the occipital cortex. Angular gyrus terminations are consistent with a possible role of the IFOF in the semantic language networks. Indeed this gyrus is highly connected through the SLF (SLF-II) to frontal regions of the inferior frontal gyrus, and dorso lateral prefrontal cortex (DLPFC) [39]. The angular gyrus is also important for spatial attention [40], [41] and spatial working memory [42]. Moreover the temporo-parietal subcomponent of the SLF connects the inferio-parietal region (including the angular gyrus) toward caudal portions of the temporal lobe involved in language function [43], [44].

The second parietal area found to be highly connected (100%) to frontal cortex through the IFOFq was the superior parietal lobe. In their paper Martino et al. refer to this connection as “the dorsal fibers of the IFOF that course in the superior portion of the sagittal stratum, along the superior portion of the lateral surface of the atrium, terminate into the convexity surface of the superior parietal lobe”. We delineated this portion of the IFOFq in all the subjects. This connection has an inferior to superior path and departs from the rest of the IFOFq, which instead runs caudally around the atrium of the lateral ventricle. Interestingly the superior parietal gyrus through the IFOFq appears to be highly connected with the premotor and prefrontal areas implicated in movement planning (Fig. 2-B). Our findings are consistent with previous literature; the superior parietal gyrus appears to have an important role in sensory motor processing and special perception [16], [45].

Our results provided also new insights into the frontal lobe areas reached by IFOFq termination. The anterior organization of the IFOFq appears to be far more complex than connections that reach just the inferior frontal gyrus and the orbitofrontal cortex. In most of the subjects (19 out of 20) a wide bilateral “fan shape” organization of the IFOFq was found to be directed towards premotor and prefrontal area of the superior frontal and middle frontal gyri (in its very rostral portion). The latter terminations showed larger inter-subjects variability. Therefore, by averaging and applying a threshold to the results on the MNI space, these terminations were not particularly evident on the 3D rendering (Fig. 1 and 2).

By combining q-ball tractography and Freesurfer parcellation we provided on a large group of individuals a consistent demonstration of Sarubbo et al. post mortem findings [15], by using a similar description of the different subcomponents of the IFOF (Fig. 6). The superficial portion towards the inferior frontal gyrus was reaching in both hemispheres the pars orbitalis and triangularis in all the subjects, whereas less frequently the pars opercularis. The IFOFq reached in all subjects the orbitofrontal cortex (deep anterior subcomponent) mainly to the lateral orbitofrontal gyrus. We delineated the deep middle component (towards the rostral middle frontal gyrus) in the whole studied group and in all subjects except one the deep posterior component (towards the superior frontal gyrus). Although the DTI results presented by Sarubbo et al. seem to confirm in vivo the post-mortem findings, there are limitations in the methodology. First, the in vivo DTI dissection was presented in just a single individual. Second, the DTI data were acquired on a different subject from the ones whose brains were dissected post mortem. Finally, DTI is nowadays an inferior tracking methodology that has been demonstrated to produce a higher rate of false results compared to our state of the art approach, in particular when used with very low FA value threshold (0.05).

The previously mentioned paper on spherical deconvolution dissection of fronto-occipital connections [25] described a “fan shape” of the anterior terminations of the IFOF very similar to the one we found with q-ball, but no detailed description was provided and no parietal termination were identified. Instead, in the same paper, the existence of a dorsal connection named superior fronto-occipital fasciculus (sFOF) was suggested. These differences with our findings can be explained by the different choices of the ROIs used for the tracking. Our seeding region (external/extreme capsule) limited our results to pathways passing through this region. From one side this allowed us to explore also the parietal terminations of the IFOFq. On the other side we couldn’t obviously evidence fronto-occipital connections not passing through this seed ROI. However as the same authors admit in their paper and based on our experience we can assume that the sFOF is likely to be a portion of the superior longitudinal fasciculus (SLF), in particular the subcomponent SLF II (from angular gyrus) rather than a new distinct pathways.

External/extreme capsule ROI based dissection approach provided a delineation of the inferior fronto-occipital fasciculus very consistent across subjects. This methodology is efficient and easily applied. This could be really important for example in brain tumor application, where the brain anatomy distortions complicate the fiber tracking results. The anterior commissure is clearly identifiable on the sagittal color map. The coronal plane passing through it, contain the external/extreme portion of the IFOFq. At this level all the IFOFq pathways gather together and pass necessary to this “gate”.

### Methodological Considerations

We are aware that diffusion modeling and fiber tracking methodology are techniques quickly developing. Therefore we believe that as novel methods become available an in-vivo delineation of the IFOF more precise and accurate will likely be possible, especially in single subjects. Within tracking methods available nowadays, we chose q-ball residual-bootstrap fiber tracking, mainly because it has been shown to perform better than either deterministic or probabilistic tracking [46] and to identify with high sensitivity and specificity pathways associated with subcortical stimulation points [31]. We applied a constant solid angle q-ball variant to model diffusion signals and find tracking directions [29], [47]; this q-ball variant resolves fiber directions at a higher resolution and with more accuracy than Tuch’s q-ball methodology [30]. We chose to use a 4^th^ order spherical harmonic in fitting data because the residual bootstrap methodology is more accurate with more degrees of freedom [48] and 4^th^ order harmonics strike a good balance between fitting the data well and preserving degrees of freedom. Although very appealing, we did not use CSD to find tracking directions because the computational times required make the combination of CSD with residual bootstrap tracking impractical.

The choice of stopping parameters (FA threshold of. 15 and a 60° angle) was based on our experience with our tracking methodology in pre-surgical planning in brain tumors on routinely acquired HARDI protocol [31]. Even though the FA threshold might be considered low, it was chosen to allow the algorithm to explore regions of low anisotropy due to the crossing of the “novel” portions of the IFOFq (bending toward the superior-middle frontal giri or the parietal lobes) with corpus callosum, corona radiata and superior longitudinal fascicle. This FA threshold was in any case much more conservative than the one previously used in the mentioned DTI publication (FA = .05) [49]. Regarding the relatively “permissive” choice of maximum angle, it might be worth noting here an important difference between residual bootstrap and probabilistic tracking. One of the issues with probabilistic methods is that when a voxel has two fiber populations, both lobes contribute to the PDF (probability distribution function) and a large angle limit exacerbates this issue. Our tracking method produces a bootstrap ODF, however only the peak closest to the incoming direction of the streamline contributes to the PDF. This means that in voxels with two or more well defined fiber populations, only one population contributes to the PDF unless the incoming angle is roughly equidistant to both fiber populations. Large angles are problematic in the absence of a co-linearity constraint based on prior identification of main diffusion directions [50]. This method of picking tracking directions serves as such a constraint and therefore mitigates the issues otherwise seen with large curvature angles.

## Conclusion

There is no convincing in vivo data published to date demonstrating both frontal and posterior termination of the IFOF. We have described an anatomy of the IFOF based on in vivo dissection by q-ball that presents a more complex structure compared to previous results with other diffusion tractography algorithms and ROI definitions. Our results suggest that the IFOF is an associative pathway that does not exclusively connect the inferior ventral portion of the inferior frontal and orbitofrontal region with the occipital lobe. In the frontal lobes our results show a wider spread of projections over the prefrontal cortex, both in the middle and in the superior frontal gyrus. Posteriorly the IFOF appears to be connected not only to occipital and occipital/temporal regions but connections toward the inferior parietal (angular and superior parietal gyri) were consistently found in all subjects. This complex anatomy of the IFOF revealed by post mortem and confirmed by q-ball tractography is compatible with a complex multifunctional role of this pathway. Other techniques such as functional imaging, magnetoencephalography or subcortical direct stimulation during awake surgery are possible candidates to further validate the existence of a more complex anatomy and functionality of the IFOF.
